# Enrichment-Free *Salmonella* Quantification in Raw Poultry at the 1 and 10 CFU/g Decision Thresholds: A Multi-Laboratory Evaluation of the Pathotrak Concentrator with Real-Time PCR

**DOI:** 10.3390/foods15193441

**Published:** 2026-09-26

**Authors:** Joseph A. Capobianco, Chin-Yi Chen, Heather Koppenhöfer, Joe Lee, Yiping He, Sean Martinos, Alexa Grace Baldwin, Jerri Lynn Pickett, Richard West, Javier Atencia

**Affiliations:** 1 Characterization and Interventions for Foodborne Pathogens Research Unit, Eastern Regional Research Center, Agricultural Research Service, United States Department of Agriculture, 600 East Mermaid Lane, Wyndmoor, PA 19038, USAheather.koppenhofer@usda.gov (H.K.); yiping.he@usda.gov (Y.H.); 2 Pathotrak Inc., Laurel, MD 20707, USA; smartinos@pathotrak.com (S.M.); abaldwin@pathotrak.com (A.G.B.); 3 OneTyson Laboratory Network Services, Tyson Foods, Inc., Springdale, AR 72762, USA; jerri.lynn.pickett@tyson.com (J.L.P.); richard.west@tyson.com (R.W.)

**Keywords:** *Salmonella*, poultry, enrichment-free detection, real-time PCR, threshold decision, multi-laboratory study

## Abstract

*Salmonella* in raw poultry drives foodborne outbreaks and recalls, and process-control decisions increasingly turn on quantitative thresholds rather than presence/absence. Acting on a threshold in real time requires accurate quantification near the cutoff and a result within one work shift. Conventional enrichment-based enumeration meets neither: it adds hours of growth before detection, and the growth step biases the count toward faster-growing, unstressed cells. Near-threshold false positives also matter: products below the limit tests positive, triggering costly and avoidable holds and recalls. We evaluated a same-shift, enrichment-free workflow that couples the Pathotrak concentrator with real-time PCR (Bio-Rad iQ-Check Salmonella II) for quantifying *Salmonella* in raw poultry, with sample preparation in about 30 min instead of 6–24 h of enrichment. The evaluation spanned 313 independent inoculations across three laboratories, three calendar years (2023–2025), and multiple seasons, technicians, instruments, and chicken sources. A latent-truth framework propagated inoculation uncertainty into sensitivity (Se) and specificity (Sp) estimates and made the half-log gray zone around each cutoff tractable. At 10 CFU/g, the workflow achieved Se 100% (20/20; 95% CI 83.2–100.0%) for samples at least 0.5 log_10_ above the threshold and Sp 94.4% (185/196; 95% CI 90.2–97.2%) below it; within the half-log gray zone, classification was Se 88.6% (95% CI 68.8–99.1%)/Sp 76.5% (95% CI 62.1–88.1%) (n = 77). In a cross-study comparison, gray-zone performance at 10 CFU/g fell within the range reported for MPN, with inoculation strain, chicken source, and plate-count method differing by site. At 1 CFU/g, the workflow returned a calibrated quantitative result within a single shift, which, to our knowledge, no enrichment-free real-time-PCR workflow has previously achieved without an incubation step. Samples more than 0.5 log_10_ CFU/g above or below a threshold were classified correctly in 205/216 (94.9%) cases at 10 CFU/g and 109/117 (93.2%; 95% CI 87.0–97.0%) at 1 CFU/g, with classification uncertainty confined to the gray zone (Se 63.2% (95% CI 49.9–76.0%)/Sp 66.5% (95% CI 54.2–77.9%), n = 166) as expected at low copy number (*Salmonella* genome copies estimated by qPCR).

## 1. Introduction

*Salmonella* is the leading bacterial cause of foodborne illness in the United States. The U.S. Centers for Disease Control and Prevention attribute approximately 1.35 million infections per year to foodborne sources [1]. Raw poultry is a primary reservoir [1,2]. Industry process-control decisions on raw poultry increasingly depend on quantitative enumeration rather than presence/absence detection. The reason is operational: an in-plant release-or-hold decision relies on whether contamination falls above or below a numeric cutoff. Two regulatory thresholds frame the current decision landscape. The U.S. Department of Agriculture, Food Safety Inspection Service (FSIS) treats 1 CFU/g as an adulteration standard for raw breaded stuffed not-ready-to-eat chicken products (89 FR 35033, finalized May 2024) [3]. FSIS finalized this standard in May 2024 but has not begun verification: after an initial six-month postponement (90 FR 15433), in December 2025 the Agency delayed implementation indefinitely, attributing the delay to accuracy limitations in available methods (including the reference MPN method) that have produced false positives at 1 CFU/g, and tying resumption to refinement of the current method or validation of a reliable alternative [4]. The standard is therefore in force but not yet subject to verification, and accurate, specific measurement near the 1 CFU/g cutoff remains an open analytical problem. A 10 CFU/g standard was proposed for raw chicken carcasses, parts, and comminuted chicken and turkey (89 FR 64678, proposed August 2024) [5]. FSIS withdrew that 10 CFU/g proposal in April 2025. A method is operationally useful at either cutoff only if it can do two things: quantify accurately at the threshold and return a result within the single work shift in which a processor must decide whether to release or hold perishable product.

Bacterial enrichment is the operational bottleneck in current *Salmonella* workflows. Culture-based reference methods, including enrichment followed by plating or most probable number (MPN), require multiple incubation steps and multi-day turnaround times. The same growth step is built into most commercial molecular detection workflows for raw poultry, where enrichment precedes the detection chemistry. In a high-throughput plant that handles perishable product, that delay translates directly into extended inventory holds and deferred release decisions.

Enrichment also introduces a serotype bias on top of the time cost. Faster-growing *Salmonella* serovars outcompete slower-growing and stress-injured cells during incubation, and the medium and selective conditions further shape which serovars dominate the post-enrichment pool [6,7,8,9]. Pre-enrichment can impose additional selective pressure, as variations in media pH can preferentially favor the recovery of certain serovars over others [10,11]. The downstream readout therefore reflects the post-enrichment population rather than the original sample composition. Two consequences follow. First, enrichment-based methods cannot reliably distinguish whether a positive sample originally contained one cell or one hundred. Second, serovars more often associated with human illness can be masked by faster-growing strains that happen to be present in the same sample [6]. Removing the enrichment step shortens the time to result and changes what the assay measures: genome copies present rather than outgrowth of culturable cells.

The Pathotrak platform is an enrichment-free sample preparation approach for raw poultry. It separates and concentrates bacteria from the food matrix using a filter-bag format and pressure-driven processing. The output is a small volume of concentrate suitable for direct molecular detection. No growth step is involved. The same procedure can also be used to recover isolates for downstream characterization.

Two prior reports established the analytical foundation for Pathotrak in raw poultry on detection chemistries other than real-time PCR. Armstrong et al. [12] coupled the Pathotrak concentrator to digital PCR (ddPCR) and demonstrated absolute quantification in a single-laboratory study (R^2^ = 0.903). Chen et al. [13] coupled the same concentrator to loop-mediated isothermal amplification (LAMP) and demonstrated multiplexed enrichment-free detection of four pathogens (*Salmonella*, *E. coli* O157, *Listeria*, and *Campylobacter*). Across both studies the same physical concentration step coupled cleanly to fundamentally different downstream chemistries. The calibration behavior in each case reflected the intrinsic precision of the downstream platform, not a limit imposed by the sample preparation step.

The present study extends the Pathotrak technology survey to real-time PCR (qPCR), the detection chemistry most widely deployed by regulatory agencies and the meat and poultry industry. We use the Bio-Rad iQ-Check Salmonella II assay as the qPCR readout. To our knowledge, this is the first multi-laboratory quantitative evaluation of iQ-Check qPCR in raw poultry under a threshold-decision framework. Real-time PCR is also the chemistry of the GENE-UP platform that FSIS adopted for *Salmonella* quantification in raw poultry rinses [14].

The natural reference point for this work is Schmidt et al. [15], the principal published multi-method *Salmonella* enumeration study in poultry. Schmidt et al. evaluated MPN, GENE-UP, BAX SalQuant, and the PiLOT real-time framework against a common set of inoculated poultry rinses and reported threshold-decision performance at 10 CFU/mL. That dataset is the benchmark against which a new enumeration workflow in raw poultry is most usefully compared. We anchor our cross-method comparison to the Schmidt et al. Table 12/Data File A10 subset, which provides one result per rinse for each comparator method. Direct enumeration comparison across studies is unit-mismatched (CFU/g of chicken tissue here versus CFU/mL of poultry rinse in Schmidt et al.). The cross-method threshold analysis is therefore performed band-relative to each method’s design threshold rather than on a shared absolute concentration scale.

Three design choices distinguish the present evaluation. First, performance is characterized using a latent-truth framework. The framework explicitly accounts for inoculation uncertainty near decision boundaries and reports both within-study enumeration accuracy and threshold-decision performance. Threshold-decision performance is the operationally relevant metric near a regulatory cutoff: a method can be moderately noisy on absolute concentration and still produce reliable above-or-below calls, which is the framing Schmidt et al. [15] also adopt. Second, the workflow is evaluated across three independent laboratories under realistic differences in instruments, operators, and sample sourcing, to characterize site-to-site calibration consistency. Third, a universal internal-control adjustment to the target Cq, derived from a pooled calibration model, is applied to improve interpretability across runs and laboratories. Performance is characterized at the 10 CFU/g and 1 CFU/g cutoffs that have featured in recent FSIS regulatory dialog for raw poultry.

The aim of the present study is therefore twofold. First, evaluate the Pathotrak + iQ-Check Salmonella II workflow as a same-shift enumeration tool at regulatory-relevant thresholds (10 CFU/g and 1 CFU/g) across three independent laboratories, characterizing both within-study enumeration accuracy and threshold-decision performance under a latent-truth framework. Second, position the resulting workflow within the broader Pathotrak methodological hierarchy that now spans digital PCR, isothermal amplification, and real-time PCR.

## 2. Materials and Methods

### 2.1. Inter-Laboratory Design and Statistical Approach

We evaluated the Pathotrak + iQ-Check Salmonella II workflow deliberately across three laboratories that differ in instruments, operators, sample sourcing, and inoculation design. Multi-site testing addresses two questions a single-laboratory study cannot: (1) whether the universal internal-control (IC)-adjusted calibration is consistent across sites in this dataset without per-site retuning, and (2) whether sites with different operational range coverage can still agree on threshold decisions in the operationally relevant sub-10 CFU/g range. The three participating sites are geographically independent and operationally distinct: the USDA Agricultural Research Service, Eastern Regional Research Center (Wyndmoor, PA, USA); the laboratory of the commercial sample-preparation supplier (Pathotrak Inc., Laurel, MD, USA; referred to throughout as the “Pathotrak Inc. site” to distinguish it from the Pathotrak platform itself); and the food-safety diagnostics laboratory of a Fortune 100 food producer (Tyson Foods, Inc., Springdale, AR, USA; referred to as “Tyson Laboratory Services”). The combined dataset was generated over a 16-month period spanning three calendar years (December 2023 through April 2025), across multiple seasons (winter, spring, and summer reactions all represented), with qPCR at each site performed by different technicians on different instruments and using chicken sourced independently by each laboratory from different commercial suppliers. Together these dimensions (geography, year, season, operator, instrument, and grower) define a robustness test that tackles the operational heterogeneity a working food-safety laboratory actually encounters. All sites followed the same Pathotrak + iQ-Check Salmonella II workflow and used standardized consumables and reagents where feasible. Chicken source, inoculation strain, and plate-count method were set by each laboratory; the concentrator, PCR assay, and decision thresholds were common to all sites.

Due to logistical and institutional constraints, chicken breast samples were sourced independently by each laboratory from local retail vendors or processing facilities. In addition, different *Salmonella* strains were used at each site, based on availability and biosafety/permission requirements. *Salmonella enterica* serovar Newport SN11/00 was used at the USDA. A *Salmonella enterica* serovar Typhimurium strain provided by NIST (unpublished) was used at Pathotrak, Inc. A 5-serovar *Salmonella* cocktail comprising *Salmonella enterica* servovars Heidelberg (wild type), Kentucky (wild type), Typhimurium (wild type), Mbandaka (wild type), and Senftenberg (ATCC #43845) was used by Tyson Laboratory Services. These design realities reflect common conditions for multi-site implementation studies and were addressed analytically by (i) using inoculation level as the primary explanatory variable and (ii) comparing laboratory effects within fixed inoculation ranges.

A total of 313 qPCR reactions were analyzed across all sites over approximately 16 months. Each reaction corresponds to one independently inoculated chicken-breast sample processed through the full Pathotrak + iQ-Check workflow; the qPCR reaction is the experimental unit throughout, and the 313 reactions therefore represent 313 independent inoculations. The study timeline was extended to accommodate site scheduling and to capture realistic sources of run-to-run variation (e.g., operator, day, instrument, and sample handling).

### 2.2. Sample Preparation and Inoculation

#### 2.2.1. Bacterial Strain Preparation

*Salmonella* strains differed by site due to availability and institutional permissions. *Salmonella enterica* serovar Newport strain SN11/00 [16] used at the USDA site was cultured by inoculating a single colony from LB agar (Becton, Dickinson and Co., Sparks, MD, USA) plates containing 50 µg/mL kanamycin (Sigma-Aldrich, St. Louis, MO, USA) into 3 mL of LB broth (LB; Becton, Dickinson and Co., Sparks, MD, USA). The culture was incubated at 37 °C with shaking at 180 RPM overnight. At the USDA laboratory, optical density was read at 600 nm (OD600) on TECAN Safire2 (TECAN, Männedorf, Switzerland) to estimate cell density, and inoculum levels were confirmed by plate count (Section 2.6). Serial tenfold dilutions in PBS were prepared by mixing 0.5 mL of culture with 4.5 mL PBS in 50 mL conical tubes.

A kanamycin-resistant *Salmonella enterica* serovar Typhimurium strain provided by NIST (unpublished) was used at the Pathotrak site. It was cultured by inoculating a single colony from TSA agar plates (Carolina, Burlington, NC, USA) containing 50 µg/mL kanamycin (Sigma-Aldrich, St. Louis, MO, USA) into 3 mL of TSB broth (Carolina, Burlington, NC, USA). The culture was incubated at 37 °C with shaking at 300 RPM overnight. At the Pathotrak Inc. laboratory, optical density was read at 500 nm (OD500) on a different spectrophotometer to estimate cell density; the wavelength differs from the USDA site because the two laboratories used different instruments, and inoculum levels were likewise confirmed by plate count (Section 2.6). Serial tenfold dilutions in TSB were prepared by mixing 100 µL of culture with 900 µL TSB in 1.5 mL vials.

At Tyson Laboratory Services, each of the 5 serovars was cultured by inoculating 10 mL of BHI broth (Becton Dickenson, Sparks, MD, USA) with a single colony picked from TSA agar plates (Becton Dickenson). Cultures were incubated statically at 36 ± 1 °C for 18–24 h. From each 10 mL culture, 0.5 mL was transferred into 40 mL of BHI broth prewarmed to 37 °C in 50 mL conical tubes, followed by incubation at 36 ± 1 °C for an additional 18–24 h. The cells were pelleted by centrifugation at 17,217× *g* for 15 min at 4 °C. The supernatant was discarded and the cells were washed twice by resuspending the pellet in 12–15 mL cold 0.85% sterile saline with vortexing (Thermo Fisher Scientific, Waltham, MA, USA) followed by centrifugation at 17,217× *g* for 15 min at 4 °C. The supernatant was discarded after each wash. The resulting cell pellets were resuspended by vortexing in 10 mL chilled 0.85% sterile saline. The 5 serovars were combined (total of 50 mL) into a sterile 90 mL specimen cup (Nalgene Nunc International Corp, Rochester, NY, USA). Serial tenfold dilutions were prepared by mixing 5 mL of the combined culture with 45 mL of chilled sterile saline in 90 mL specimen cups. To establish the target inoculum concentration, serial dilutions were spread plated as described in Section 2.6 and incubated at 36 ± 1 °C for 18–24 h to permit colony development. The cultures were held at 4 °C overnight until colony-forming units (CFUs) were enumerated from the spread plates at which point calculations were performed to obtain the required inoculation concentration.

#### 2.2.2. Chicken Source and Handling

Boneless, skinless chicken breasts were obtained from local retail stores or processing facilities at each study site and processed upon receipt. Chicken was cut into large chunks using sanitized tools. For each sample, 325 g of tissue was placed into a sterile Pathotrak bag (Pathotrak Inc., Laurel, MD, USA). Bag openings were wiped clean with Kimwipes (Kimberly-Clark, Roswell, GA, USA) before heat-sealing to prevent leakage.

#### 2.2.3. Individual Sample Inoculation

To avoid heterogeneous distribution associated with bulk inoculation and subsampling, each 325 g of poultry sample was inoculated individually. Inoculation levels spanned approximately two orders of magnitude around operationally relevant thresholds (including 1 CFU/g and 10 CFU/g), depending on the study phase and site. Inoculum volumes were calculated from culture dilutions to deliver the desired CFU/g to a 325 g sample mass. Inocula were applied to the meat surface by pipetting through the bag port. After inoculation, samples were held at 4 °C for up to two hours before assay processing to allow bacterial attachment to the meat surface and were then processed within a controlled time window to minimize growth-related drift. This surface-attachment step is a deliberate feature of the study design: it ensures the workflow is evaluated on its capacity to recover and detect surface-attached cells with the operationally relevant population in raw-poultry process control rather than on detection of cells spiked directly into a rinse (cf. Section 4.4).

### 2.3. Pathogen Separation and Concentration (Pathotrak)

Following the surface-attachment hold described in Section 2.2.3, each Pathotrak bag received 300 mL of reconstituted Pathotrak Poultry Rinse Buffer (Pathotrak Inc., Laurel, MD, USA); the rinse and subsequent Pathotrak concentration steps therefore evaluate the combined efficiency of releasing surface-attached cells into the rinse and recovering them from that rinse, rather than detection of cells spiked directly into a rinse. The buffer was supplied as a concentrate and reconstituted according to the manufacturer’s instructions by adding 4 L of sterile nanopure water (Thermo Fisher Scientific, Waltham, MA, USA). Bags were mixed for 10 s to 1 min (manual massage or belly dancer shaker; Stovall Life Science, Greensboro, NC, USA).

Air was expelled, and the Pathotrak filter assembly unit was attached and sealed. Bags were then loaded into Pathotrak Pods (Pathotrak Inc.), and up to five samples were processed per cycle for 10 min using the manufacturer’s preset isostatic pressure program.

### 2.4. Elution and Concentrate Handling

After processing, the Pathotrak concentrator (Pathotrak NGE-EXTR-3) was removed and backwashed with 1 mL phosphate-buffered saline (PBS; Sigma-Aldrich, St. Louis, MO, USA) using a 3 mL syringe (BD Biosciences, Franklin Lakes, NJ, USA). The backwash was delivered in two 0.5 mL aliquots. For USDA only, it was followed by a 3 mL air push to maximize backflush recovery. The air-push step was an in-house procedural variation: it was used at all three sites in early testing but was discontinued at the Pathotrak Inc. and Tyson Foods sites partway through the study to simplify the workflow for routine high-throughput operation, accepting a small, expected reduction in low-end recovery sensitivity in exchange. The change was not propagated to the USDA site for the duration of data collection, and the USDA dataset therefore reflects the air-push protocol throughout. We report both versions of the protocol in full because the procedural difference is a candidate contributor to the site-level intercept shift discussed in Section 4.3. The eluate was collected in 1.5 mL microcentrifuge tubes (Eppendorf, Hamburg, Germany) and centrifuged at 12,000× *g* for 1 min (Eppendorf 5424 or equivalent).

Supernatants were reduced to approximately 20 µL using the Pathotrak automated pipettor (Pathotrak Inc.). Pellets were resuspended by vortexing (VWR, Radnor, PA, USA), followed by a brief spin prior to lysis.

### 2.5. qPCR Detection

The entire 20 µL concentrated eluate was lysed directly by adding 40 µL of Reagent A (Bio-Rad Laboratories, Hercules, CA, USA) and incubating at 95 °C for 15 min with shaking (1300 rpm) in a dry bath shaker (Eppendorf Thermomixer^®^ R Dry Block Heating and Cooling Shake-EPP 022628001). Lysates were centrifuged at 12,000× *g* for 2 min.

The qPCR master mix was prepared according to the iQ-Check™ Salmonella II User Manual [17]. Reactions were assembled with 45 µL master mix and 10 µL lysate in 96-well plates (Bio-Rad) and amplified on a CFX96 Deep Well Real-Time PCR System paired with C1000 Touch Thermal Cycler (Bio-Rad) using CFX Manager software (4.3.016.0321) with the manufacturer-recommended cycling program. The assay includes an internal amplification control (IAC).

For each reaction, the following values were recorded when available: (i) Salmonella target Cq and (ii) IAC Cq. Reactions with no measurable target amplification by cycle 45 were classified as non-detects (“undetermined” target) per manufacturer’s instruction. Non-detects are reported as method-negative outcomes for all downstream analyses (see Section 2.8 for handling regression fits and Section 2.9 for handling threshold analyses).

### 2.6. Plating for Enumeration

The *Salmonella* inoculum concentration was quantified using microbial plate counts. Aliquots of the inoculum suspension were plated onto LB agar supplemented with 50 µg/mL kanamycin (USDA and Pathotrak) or TSA (Tyson) and incubated at ~37 °C overnight. At the USDA site, replicate plating used the 6 × 6 drop-plate method [12]; the other two sites used standard spread-plating of 100 µL serially diluted samples in triplicate. In all cases, plate counts were used to estimate the mean and standard deviation of CFU/g for each inoculation condition. Plate-count means and SDs were carried into the latent-truth threshold analysis (Section 2.9).

### 2.7. Internal-Control Adjustment of Target Cq (IC-Adjusted Cq)

To account for run-associated Cq shifts captured by the internal amplification control (IAC), an internal-control-adjusted target cycle threshold was calculated for each qPCR reaction. Target Cq ($C_{q,T}$) and IAC Cq ($C_{q,IAC}$) were recorded from the same well. The IC-adjusted endpoint was computed as:

$$C_{q,adj}=C_{q,T}-\beta_{IAC}\;(C_{q,IAC}-33.49)$$

where $\beta_{IAC}$ is the universal IAC coupling coefficient estimated from the pooled calibration model (Section 2.8; estimated value 0.437, reported with full coefficient table in Section 3.1) and 33.49 is the IAC reference cycle, taken as the pooled mean IAC Cq across all reactions in the multi-site dataset. The reference value is fixed (not fitted) and serves only to center the IAC covariate, $C_{q,IAC}\;-\;33.49$, so that a reaction with IAC Cq equal to the reference receives no adjustment. In this formulation, reactions with later-than-reference IAC amplification ($C_{q,IAC}$ > 33.49) are adjusted downward (earlier), and reactions with earlier-than-reference IAC amplification ($C_{q,IAC}$ < 33.49) are adjusted upward (later). The adjustment was applied only to reactions with measurable target Cq and measurable IAC Cq.

### 2.8. Statistical Analysis

Target Cq and IAC Cq were recorded for each qPCR reaction (total dataset n = 313 inoculated reactions across three laboratories: 285 detected and 28 non-detects). Non-detects (defined in Section 2.5) were excluded from regression calibration fits and retained as method-negative outcomes in threshold analyses. Each run included one uninoculated negative control, processed identically to the inoculated samples; all negative controls returned no amplification and are excluded from the inoculated-sample denominators reported throughout.

A pooled ordinary least squares (OLS) regression was fit to detected reactions using target Cq as the dependent variable and ${log}_{10}$ (inoculation level, CFU/g) and IAC deviation from the reference value (33.49 cycles) as predictors:

$$C_{q,T}=\beta_{0}+\beta_{1}{log}_{10}\left(\mathrm{CFU}/g\right)+\beta_{IAC}(C_{q,IAC}-33.49)$$

Model coefficient estimates are reported in Results. For plotting and thresholding, the fitted model was expressed on the IC-adjusted endpoint $C_{q,adj}$ defined in Section 2.7.

To assess laboratory (site) effects, a pooled model including laboratory indicator terms (Pathotrak as reference) was fit separately, of the form

$$C_{q,T}=\beta_{0}+\beta_{1}{log}_{10}\left(\mathrm{CFU}/g\right)+\beta_{IAC}(C_{q,IAC}-33.49)+\gamma_{Tyson}\cdot 1\{\mathrm{lab}=\mathrm{Tyson}\}+\gamma_{USDA}\cdot 1\{\mathrm{lab}=\mathrm{USDA}\}$$

Here $\gamma_{Tyson}$ and $\gamma_{USDA}$ are site intercept shifts in cycles relative to the Pathotrak reference site, and 1{lab = X} is an indicator equal to 1 for reactions from laboratory X and 0 otherwise. Coefficient estimates with standard errors (SEs) and per-site intercept shifts are reported in Section 3.1. Per-laboratory regression lines and confidence bands shown in Figure 1 are descriptive only; inference regarding laboratory shifts is based on the pooled lab-effects model.

### 2.9. Threshold Performance Using a Latent-Truth Framework

Threshold performance was evaluated at inoculation thresholds of 10 CFU/g and 1 CFU/g using a latent-truth framework [18,19] to account for *Salmonella* cell number uncertainty near each decision point. For each inoculation condition, plating-derived mean and standard deviation on the linear concentration scale were converted to log-normal parameters [20,21] and used to estimate the probability that the true concentration exceeded the threshold T, denoted $p_{true}$. Test positivity within each concentration band was modeled as:

$$P(test+)=p_{true}\;Se+(1-p_{true})(1-Sp)$$

where *Se* is sensitivity and *Sp* is specificity. This is the standard relationship between apparent and true positivity for a single imperfect test [22]. Identifiability here comes from the known inoculation distribution, which supplies p_true directly, rather than from multiple tests applied to the same samples as in conventional latent-class designs. Sensitivity and specificity were estimated by maximum likelihood within bins defined by ${log}_{10}$ distance from the threshold: >+0.5, +0.25 to +0.5, ±0.25 (gray zone), −0.25 to −0.5, and <−0.5. The ±0.25 log_10_ width is half the ±0.5 log_10_ (half-log) tolerance applied to quantitative Salmonella results in the FSIS-proposed Salmonella Framework for Raw Poultry Products (withdrawn in 2025), lies at the upper end of the reproducibility standard deviations reported for colony-count methods (about 0.1–0.3 log_10_; e.g., the worked examples in ISO 19036:2019 [23]) and is roughly three times the pooled replicate standard deviation of the inoculum plate counts in this study (≈0.09 log_10_ across 29 inoculation levels; 0.04–0.13 by site). Non-detect target outcomes (no measurable target Cq by cycle 45) were treated as method-negative results in the latent-truth threshold analysis. Above- and below-threshold operating points are reported with Clopper–Pearson exact 95% confidence intervals [24]; gray-zone sensitivity and specificity, estimated jointly by maximum likelihood under the latent-truth model, are reported with profile-likelihood 95% intervals [25] confirmed by nonparametric bootstrap (4000 resamples) [26]. Clopper–Pearson intervals for the above- and below-threshold bands are provided for all methods in Supplementary File S1; profile-likelihood gray-zone intervals are provided for the Pathotrak workflow.

For cross-method threshold comparisons with Schmidt et al. [15], only the 10-threshold comparison was used because that study was designed around identification of poultry rinses with *Salmonella* concentrations ≥10 CFU/mL. The primary direct-comparison Schmidt subset was Table 12/Data File A10, which provides one result per rinse (n=101) and uses only the A technical repeat for GENE-UP and BAX. Repeat-level and repeat-averaged GENE-UP/BAX datasets were excluded from the main comparison to avoid pseudo-replication and inflation of fit metrics through averaging.

Enumeration performance was summarized within study using slope, intercept, $R^{2}$, root-mean-square error (RMSE), and quantification discrepancy on the ${log}_{10}$ scale. For Pathotrak, enumeration summaries were computed using leave-one-laboratory-out cross-validation on detected reactions. Calibration curves from different studies were not overlaid as directly interchangeable because workflows, matrices, and reporting units differed (CFU/g versus CFU/mL); comparisons were therefore reported in terms of within-study error structure and bias.

## 3. Results

Headline result. Across three independent laboratories and 313 qPCR reactions, the Pathotrak + iQ-Check workflow delivered sensitivity (Se) = 100% [95% CI 83.2–100.0%] (n = 20) for samples ≥0.5 log_10_ above the 10 CFU/g decision threshold (>31.6 CFU/g) and specificity (Sp) = 94.4% [95% CI 90.2–97.2%] (n = 196) for samples ≥0.5 log_10_ below the threshold (<3.16 CFU/g); the above- and below-threshold operating bands are directly comparable to the published method comparator [9]. Within the ±0.25 log_10_ gray zone immediately surrounding the cutoff (5.62–17.8 CFU/g), classification was Se 88.6% [95% CI 68.8–99.1%]/Sp 76.5% [95% CI 62.1–88.1%] (n = 77). Calibration was consistent in slope and IAC coupling across the three sites. The supporting calibration analysis, between-site agreement evidence, within-study enumeration accuracy, and 1 CFU/g threshold characterization are reported in the subsections that follow. Clopper–Pearson intervals for the remaining above- and below-threshold bands (all methods) and profile-likelihood gray-zone intervals (Pathotrak) are tabulated in Supplementary File S1 (sheets Latent_threshold10_all_methods and Latent_threshold1_Pathotrak).

### 3.1. Descriptive Per-Laboratory Dose–Response After Universal Internal-Control Adjustment

To summarize the within-laboratory dose–response relationship after applying the universal internal-control adjustment, the IC-adjusted target Cq ($Cq_{adj}$) was evaluated as a function of inoculation level separately for each participating site. Across the three sites (Tyson Laboratory Services, Pathotrak Inc., and USDA), $Cq_{adj}$ decreased significantly with increasing inoculation level consistent with expected qPCR behavior. Figure 1 shows these fits by site—(A) Tyson, (B) Pathotrak, and (C) USDA—on shared axes, so intercept differences between laboratories are visible by inspection.

Regression fit statistics are summarized in Table 1. Although slopes were broadly similar across laboratories, fitted intercepts differed; in particular, USDA exhibited an intercept shift relative to the other sites (Table 1; Figure 1C). Slopes are directionally consistent across all three sites (negative, similar magnitude). The Tyson R^2^ = 0.35 reflects a narrow predictor range (no observations above 10 CFU/g; seven unique levels) rather than degraded assay performance. The mechanism for this shift is examined in Section 4.2. Non-detect target outcomes (no measurable target Cq by cycle 45) were excluded from the regression fits summarized here but were retained as method-negative outcomes in the threshold analyses described later in Section 3.2 and Section 3.3.

Pooled universal model. After fitting the pooled OLS model in Section 2.8 to all 285 detected reactions across the three laboratories, the estimated coefficients were β0 = 37.738 (SE 0.122), β1 = −3.178 (SE 0.157, *p* = 8 × 10^−57^), and β_IAC_ = 0.437 (SE 0.060, *p* = 3 × 10^−12^). The IAC term is highly significant, supporting the universal IC adjustment used throughout this paper. In leave-one-site-out refits, β_IAC_ was 0.484 with USDA held out, 0.390 with Pathotrak held out, and 0.489 with Tyson held out, against 0.437 on the full data (Supplementary File S1). The full model is Target Cq = 37.738 − 3.178 · log_10_(CFU/g) + 0.437 · ($Cq_{IAC}$ − 33.49).

Laboratory effects. Refitting the pooled model with laboratory indicator terms (Pathotrak as reference) yielded a non-significant Tyson shift (γ_Tyson_ = −0.219 cycles, SE 0.203, *p* = 0.28) and a large, highly significant USDA shift (γ_USDA_ = −2.044 cycles, SE 0.221, *p* = 6 × 10^−18^). Coefficient estimates from this model are summarized in Table 2. The slope and IAC coupling were unchanged in sign and magnitude (β1 = −2.985, SE 0.147; β_IAC_ = 0.519, SE 0.052), indicating that the USDA intercept shift reflects a vertical offset in the calibration rather than a different concentration–response relationship. This pattern is consistent with a front-end recovery difference, plausibly the alternative backflush procedure used at the USDA site (Section 2.4); implications are discussed in Section 4.2.

**Table 2:** Pooled multi-site calibration model with laboratory indicator terms. Model fit by ordinary least squares to all 285 detected reactions; Pathotrak is the reference laboratory. γ_Tyson_ and γ_USDA_ are intercept shifts relative to Pathotrak. The IAC term centers on the pooled mean IAC reference of 33.49 cycles.

Term	Estimate	SE	*p*-Value
Intercept (Pathotrak ref.)	38.198	0.192	<10^−300^
log_10_(CFU/g)	−2.985	0.147	5 × 10^−57^
Cq_IAC_ − 33.49	0.519	0.052	4 × 10^−20^
Tyson shift (γ_Tyson_)	−0.219	0.203	0.28
USDA shift (γ_USDA_)	−2.044	0.221	6 × 10^−18^

### 3.2. Between-Site Agreement in the Operational Sub-10 CFU/g Range

Because the inoculation series differed by site, between-laboratory agreement on threshold calls at 10 CFU/g could not be evaluated directly across all three sites; the cleanest within-study comparison is the overlap window where the Tyson and Pathotrak Inc. sites both have data, which spans approximately 0.8–9.6 CFU/g (entirely sub-threshold relative to a 10 CFU/g cutoff) (Table 3). Within that window, qPCR detection rates were essentially identical between the two sites: 84.0% (Pathotrak Inc., n = 25) versus 82.5% (Tyson, n = 126) in the low sub-band (≤1.5 CFU/g; Δ = 1.5 percentage points; Fisher exact *p* = 1.00), and 100.0% (Pathotrak Inc., n = 10) versus 100.0% (Tyson, n = 38) in the high sub-band (5–10 CFU/g; Δ = 0.0 percentage points; Fisher exact *p* = 1.00).

Within-overlap regression of IC-adjusted Cq on log_10_(CFU/g) with a laboratory indicator (detected reactions only, *n* = 187) yielded a Tyson-versus-Pathotrak Inc. site offset of −0.50 cycles (SE 0.23, *p* = 0.033) and a slope of −2.44 (SE 0.23). Dividing the 0.50-cycle offset by the fitted slope (|−2.44| cycles per log_10_ CFU/g) converts it to approximately 0.20 log_10_ CFU/g in back-calculated concentration. This is smaller than the half-log gray-zone width (±0.25 log_10_) used in the threshold-decision framework. The two sites would therefore be expected to agree on which side of the gray zone a near-threshold sample falls, even though the within-window inoculation-adjusted offset is statistically detectable. We note that the Tyson × Pathotrak Inc. site overlap window is entirely below the 10 CFU/g threshold (0.8–9.6 CFU/g), so this between-site agreement statement is an inference from the magnitude of the inoculation-adjusted offset rather than a direct observation of agreement on at-threshold or above-threshold samples. We note that this within-window offset is detectable here; whereas, the broader pooled lab-effects model (Table 2) reported the Tyson term as not significant; the two estimates address different ranges of the data, and the within-window estimate is the more relevant one for inferring threshold-call agreement in the operational sub-10 CFU/g band.

### 3.3. Latent-Truth Threshold Performance at the 10 CFU/g Threshold

Threshold-decision performance at the 10 CFU/g threshold was evaluated using the latent-truth framework described in Methods. This estimates sensitivity and specificity within concentration bands defined as fixed offsets from the threshold. For direct cross-method comparison, Pathotrak results were evaluated relative to 10 CFU/g, whereas the Schmidt et al. dataset was evaluated relative to 10 CFU/mL; performance was compared within matched relative bands rather than by forcing a shared absolute concentration scale.

Figure 2 summarizes band-specific threshold performance for Pathotrak alongside the Schmidt et al. comparator methods (full numerical values are tabulated in Supplementary Table S1 for the cross-method enumeration comparator and as Figure 2 cell labels for the band-specific Se/Sp values). Bands are defined by relative log_10_ distance from each method’s threshold rather than by an absolute concentration scale: Pathotrak bands are relative to 10 CFU/g of chicken tissue, and Schmidt bands are relative to 10 CFU/mL of poultry rinse. Matrices, units, and front-end workflows differ between studies, so the side-by-side display characterizes whether each method discriminates near its own design threshold; it is not a head-to-head ranking at a fixed absolute concentration.

In the gray zone (±0.25 log_10_ around the threshold), Pathotrak exhibited a balanced classification profile: Se 88.6% [95% CI 68.8–99.1%] and Sp 76.5% [95% CI 62.1–88.1%] (n = 77); a side-by-side bar comparison of gray-zone Se and Sp across all four methods is provided in Figure 3. For this comparison we adopt 75% on both sensitivity and specificity as a pragmatic lower bound for balanced near-threshold performance: a method clearing 75% on both axes neither misses above-threshold product nor over-calls below-threshold product at a rate that would dominate operational decisions. The 75% value is a descriptive reference for comparing operating-point balance across methods, not a regulatory acceptance criterion or a validated cutoff.

Specificity was high in the below-threshold bands: Sp 94.4% [95% CI 90.2–97.2%] at <3.16 CFU/g and Sp 90.0% at 3.16–5.62 CFU/g. Sensitivity increased with distance above the threshold: Se 80.0% at 17.8–31.6 CFU/g and Se 100.0% at >31.6 CFU/g.

Across comparator methods, MPN showed similar gray-zone balance (Se 76.8%; Sp 76.5%), whereas GENE-UP exhibited reduced gray-zone specificity (Sp 20.8%) and BAX exhibited reduced gray-zone sensitivity (Se 8.1%) despite high specificity in that band (Sp 100.0%).

PiLOT operating points are not shown in Figure 2 because PiLOT is a binary threshold test rather than an enumeration method. PiLOT (Poisson Limit One Tube), introduced by Schmidt et al., calls a positive or negative at a fixed concentration threshold from the presence or absence of Salmonella in a single enrichment tube. The four PiLOT variants reported in Schmidt et al. (PiLOT-95, -86, -63, -50) differ in the assay volume. The volume sets the Poisson probability of a positive test at the 10 CFU/mL design threshold (0.95, 0.86, 0.63, 0.50, respectively). Because the test is binary by construction, it retains no quantitative information about the initial concentration and cannot be used for enumeration. It is therefore not a direct comparator for the enumeration analysis in this section or Supplementary Table S1. Full PiLOT Se/Sp values are tabulated in Supplementary Figure S1 for completeness.

Interpretation of gray-zone performance and method-by-method patterns is provided in Section 4.

Within-study enumeration accuracy is reported here briefly as supporting context. On the leave-one-laboratory-out cross-validation across all 285 detected reactions, Pathotrak + qPCR was nearly unbiased relative to plating (slope 1.06, intercept −0.05, mean log_10_ quantification discrepancy ≈ −0.02; RMSE 0.57 cycles). Comparator method metrics from Schmidt Table 12/Data File A10 (MPN, GENE-UP, BAX) are tabulated in Supplementary Table S1; because the comparator dataset is calibrated in CFU/mL of poultry rinse rather than CFU/g of chicken tissue, those metrics are not directly substitutable for the Pathotrak values and the threshold-decision comparison above is the more conservative cross-study read.

### 3.4. Latent-Truth Threshold Performance at the 1 CFU/g Threshold (Pathotrak Dataset Only)

The 1 CFU/g threshold corresponds to the active U.S. FSIS adulteration standard for raw breaded stuffed not-ready-to-eat chicken products (89 FR 35033) [3]. Pathotrak + iQ-Check is the only enrichment-free real-time-PCR workflow we are aware of that quantifies at this threshold without an additional centrifugation-based concentration step. Latent-truth threshold performance at 1 CFU/g was evaluated for the Pathotrak dataset only because the Schmidt direct-comparison study was designed around the 10 CFU/mL threshold. Per-band Pathotrak performance at 1 CFU/g is summarized below and shown in Figure 4.

As expected, classification in the near-threshold region was more difficult at the 1 CFU/g decision point than at the 10 CFU/g threshold. In the gray zone (±0.25 log_10_ around 1; 0.562–1.778 CFU/g), Pathotrak exhibited Se 63.2% [95% CI 49.9–76.0%] and Sp 66.5% [95% CI 54.2–77.9%] (n = 166). Performance improved with increasing distance above the threshold (Se 96.0% for 1.778–3.162 CFU/g, n = 25; and Se 93.2% [95% CI 87.0–97.0%] for >3.162 CFU/g, n = 117). In the modestly below-threshold band (0.316–0.562 CFU/g), specificity was 100.0% (n = 5). No observations were available in the far-below band (<0.316 CFU/g), reflecting study coverage rather than model behavior. Interpretation of low-copy behavior at the 1 CFU/g decision point is provided in Section 4.

## 4. Discussion

### 4.1. Threshold-Decision Performance

At the 10 CFU/g decision point, Pathotrak + qPCR was among the most balanced methods in the gray zone, providing a practical trade-off between missing above-threshold samples and over-calling below-threshold samples. Together with the high specificity observed at well-below bands and the high sensitivity observed at well-above bands, this pattern supports use of the workflow as a same-shift threshold-screening assay near 10 CFU/g. MPN and Pathotrak + qPCR are both enumeration methods: each returns a quantitative concentration estimate that supports threshold decisions and post hoc operational analysis. The PiLOT variants, by contrast, are binary threshold tests that do not retain quantitative information about the initial concentration. The threshold-decision performance reported above is therefore one of two capabilities Pathotrak offers, alongside enumeration through the calibrated Cq endpoint.

At the 1 CFU/g decision point, gray-zone classification was materially more difficult (Se 63.2%, Sp 66.5%; n = 166). This is the expected behavior near a low-copy threshold. It reflects the sampling statistics of low-copy detection, not a workflow-specific deficit. At the 1 CFU/g decision point each 325 g sample carries only a few hundred target cells, and recovery losses and the 1:6 lysate split (10 µL of a 60 µL lysate, Section 2.5) reduces that number further before amplification. Poisson sampling at recovery, the inherent partition-uncertainty of qPCR amplification, and plating-derived inoculum dispersion therefore all contribute variance on a comparable scale to the half-log gray-zone width itself. Small differences in any of these terms shift a replicate from just above to just below the decision boundary. The latent-truth framework is the appropriate analysis here. It models each near-threshold sample’s reference status as a probability rather than forcing a binary above/below call from the plated mean alone.

An enrichment-based alternative at 1 CFU/g would compress the concentration axis into a binary detected/not-detected outcome and add 18–24 h of incubation and second-day handling. Enrichment-based workflows cannot quantitatively distinguish 1 CFU/g from 100 CFU/g in a single result because growth saturates the signal long before readout [9,27]; whereas, the Pathotrak + qPCR workflow reported here returns a calibrated quantitative estimate at the 1 CFU/g threshold within a single work shift, which, to our knowledge, commercial enrichment-dependent platforms cannot reach without additional incubation. The Pathotrak gray-zone Se 63.2%/Sp 66.5% is therefore a measured low-copy uncertainty in a workflow that retains quantitative information and same-shift turnaround, not a benchmark against a perfect reference.

Specificity is the structural advantage of an enrichment-free workflow. Industry decisions depend on both false negatives (which release contaminated product) and false positives (which trigger expensive holds and recalls), and enrichment-based methods cannot deliver high specificity at low concentrations because growth during incubation amplifies any background signal that survives matrix extraction [27]. Skipping the growth step removes that source, and the resulting specificity gap appears clearly in the present comparison: Pathotrak + qPCR at 10 CFU/g (Se 88.6%, Sp 76.5%) shows gray-zone performance within the range reported for MPN in a cross-study comparison at 10 CFU/mL (Se 76.8%, Sp 76.5%; Figure 3), while the GENE-UP and BAX comparators show structurally different operating-point biases (sensitivity-leaning and specificity-leaning, respectively; values in Figure 3). At the ten-fold harder 1 CFU/g threshold, Pathotrak + qPCR delivered Se 63.2%/Sp 66.5% in the ±0.25 log_10_ gray zone, showing balanced performance at a regulatory cutoff that the centrifugation + GENE-UP comparator in [15] reaches only with enrichment. The comparison is band-relative because this study measures CFU/g of chicken tissue while Schmidt et al. measure CFU/mL of poultry rinse. The unit-basis difference and its consequences for cross-method comparison are addressed in Section 4.3 and Section 4.4.

Specificity near the threshold is the dominant analytical challenge for any 1 CFU/g method, and it is a producer concern as much as a regulatory one: false positives (product that is in fact below the limit testing positive) drive wrongful holds and recalls. FSIS has noted that false positives at low contamination levels are a limitation of currently available methods [4]. Because the present workflow omits the enrichment step that amplifies residual background signal, it directly targets that false-positive failure mode and characterizes operating-band specificity at the 1 CFU/g decision point rather than inferring it from presence/absence data.

### 4.2. Error Stack and Methodological Hierarchy

The performance gap between this Pathotrak + qPCR workflow and conventional culture-based enumeration is best understood as a sequential elimination of error sources. Conventional enrichment-based detection of *Salmonella* in raw poultry combines two compounding error terms. The first is an exponential bacterial-growth phase during incubation. The second is an exponential DNA-amplification phase during qPCR readout. During incubation, faster-growing serovars and unstressed cells are over-represented and slower- or stressed-cell populations are under-represented [6]. The resulting bias is strain- and condition-dependent and cannot be removed analytically. The Pathotrak platform removes the first term entirely. Bacteria are physically separated and concentrated from the matrix without an incubation step, so the read-out reflects the initial cell load on the sample rather than the post-incubation amplified load.

Coupling the Pathotrak concentrator to a digital partition-based readout additionally removes the second exponential term. Examples include ddPCR, as used by Armstrong et al. [12], and emerging partition-based qPCR formats such as nanoplate qPCR architectures now reaching the commercial market. In a partition-based assay each reaction is independent and the readout is a count of positive partitions, so the relationship between input copies and measured signal is linear rather than exponential. Coupling the Pathotrak concentrator to a real-time qPCR readout as presented in the present work eliminates the first exponential term but retains the second, because qPCR quantification still depends on the cycle threshold of an exponential amplification curve. Even with the DNA-amplification term still present, Pathotrak + qPCR achieves gray-zone classification balance at 10 CFU/g (Se 88.6%, Sp 76.5%) within the range reported for MPN in a cross-study comparison. MPN is the established enumeration reference [15]. One hypothesis consistent with this observation is that the growth step contributes much of the variance of enrichment-based methods; testing it would require a within-study comparison of the same samples with and without enrichment, which is outside the scope of this study.

These observations support a methodological hierarchy rather than a winner–loser comparison among Pathotrak-based pairings. Pathotrak + ddPCR (and analogous partition-based platforms) provide absolute, partition-counting linear quantification at a higher operational cost, a more involved workflow, longer total turnaround (on the order of several hours), more expensive instrumentation, and a quantification range that is bounded at the low end by the droplet (or partition) input requirement. Armstrong et al. [6] report that reliable quantification requires a minimum of 10,000 accepted droplets per reaction, with quantification demonstrated across the 1–50 CFU/g range. Pathotrak + qPCR, by contrast, completes the post-concentration detection workflow in approximately one hour, retains usable detection (rather than absolute quantification) below 1 CFU/g, and produces threshold-decision performance within the range reported for MPN in a cross-study comparison. The two pairings are therefore complementary, not competing. Partition-based platforms are well suited as gold-standard reference instruments for absolute quantification and method-validation studies. They are candidates for the roles currently occupied by MPN. Real-time qPCR coupled to the Pathotrak concentrator is well suited for routine high-throughput screening and threshold-decision deployment in operational food-safety settings. Same-shift turnaround and integration with widely available qPCR infrastructure are the dominant operational constraints in those settings, including the FSIS-adopted GENE-UP platform [14,15].

### 4.3. Site Effects: USDA Intercept Shift and Tyson Design Range

Two site-level patterns warrant discussion. First, USDA exhibited a calibration intercept shift of approximately −2.0 cycles relative to Pathotrak (Table 2), with no corresponding change in slope or in the IAC coupling. One plausible explanation is the alternative USDA backflush protocol. That protocol adds a 3 mL air push after the two-aliquot PBS backwash (Section 2.4). It likely increases recovery efficiency relative to the liquid-only backflush used at the Pathotrak Inc. site. The vertical offset without slope change is consistent with this hypothesis. Internal bench-scale work conducted by the workflow developer (Pathotrak Inc., unpublished) is directionally consistent with this hypothesis, showing a measurable recovery improvement when the air-push step is included. The air-push step is one candidate explanation for this shift; the inoculation strain and its resistance marker, culture conditions (shaking at USDA and Pathotrak, static at Tyson), chicken source, and plate-count method also differ by site and cannot be separated in this design. Since the present study did not isolate the air-push effect in a controlled experiment, we treat the recovery-step explanation as a candidate hypothesis for the focused future study described in Section 4.6 rather than an established cause. The air-push is therefore an optional sensitivity-enhancing step rather than the default protocol, since it is not readily automated in the standard high-throughput configuration. The operational implication is that the workflow is deployable across sites with materially different recovery-step procedures because the universal IC-adjusted endpoint Cq_adj_ used throughout this paper provides a common calibration anchor. Without IC adjustment, a −2 cycle site shift would translate to roughly two-thirds of a log in apparent concentration. That gap is larger than the half-log gray-zone width itself and would preclude transferring calibration constants between sites. With IC adjustment, the slope and IAC coupling are preserved across sites (Table 2). Threshold-decision performance is consistent across the multi-site dataset (Section 3.3). The residual intercept difference therefore becomes a tractable site covariate rather than a barrier to deployment. Identifying the procedural source of the shift remains scientifically useful and is the subject of the focused recovery-step study described in Section 4.6, but it is not a precondition for operational use of the workflow.

Second, the lower within-laboratory R^2^ observed at Tyson (R^2^ = 0.35; Table 1) should be interpreted as a design-range artifact rather than as evidence of poor assay performance. The Tyson inoculation series spans approximately 0.8 to 9 CFU/g across only seven unique levels, with no observations above the 10 CFU/g threshold, whereas the Pathotrak Inc. site (16 levels, 0.36–62.77 CFU/g) and USDA (six levels, 1.41–62.91 CFU/g) span more than two log units. A narrower predictor range mathematically depresses R^2^ even when slope and residual variance are comparable, and the Tyson slope estimate (Table 1) is in fact directionally consistent with the other sites. To verify this directly, we refit the Pathotrak Inc. and USDA dose–response data after truncating each to the Tyson concentration range (0.8–9 CFU/g). Pathotrak Inc.’s R^2^ fell from 0.74 (full range, n = 74) to 0.58 (truncated, n = 29), and USDA’s fell from 0.72 (full range, n = 69) to 0.16 (truncated, n = 40), the latter reflecting the fact that USDA’s inoculation design placed almost no points between 1.83 and 9 CFU/g, so restricting to Tyson’s range colm lapses its dynamic range entirely. Both sites’ R^2^ drop substantially under the same range restriction, confirming that the lower Tyson R^2^ is driven by the inoculation design rather than by site-level assay performance. The Tyson dataset remains useful for evaluating reproducibility within the operationally relevant band immediately below the 10 CFU/g decision point, but it is not informative about high-concentration linearity.

### 4.4. Cross-Method Enumeration, Unit Caveats, and the GENE-UP Protocol-Level Comparison

Within-study enumeration metrics (Supplementary Table S1) place the Pathotrak + qPCR workflow as nearly unbiased relative to plating (slope 1.06, mean QD ≈ −0.02 log_10_ in the LOLO summary) but noisier than MPN, which remains the most precise enumeration comparator on the Schmidt A10/Table 12 dataset (R^2^ = 0.91). GENE-UP’s compressed slope (0.69) and BAX’s systematic offset (mean QD ≈ −1.18 log_10_) are visible in the same table. These rankings should be interpreted within their native unit and matrix. The Schmidt et al. [15] design inoculates the rinsate directly. Bacteria were introduced into approximately 400 mL of carcass rinse and evaluated already in suspension. The CFU/mL threshold therefore characterizes detection of free-floating cells. The present study inoculates 325 g chicken-breast cubes. Inoculated cubes are held for at least two hours to allow surface attachment. The Pathotrak rinse and concentration steps are then applied. The CFU/g threshold therefore characterizes recovery plus detection of surface-attached cells. This is the operationally relevant quantity for in-plant decisions on raw product. The two designs evaluate adjacent but non-identical physical processes. A simple mass-to-volume conversion would assume 100%-efficient rinse extraction from a homogeneously contaminated surface. That assumption is exactly what the surface-attachment design is meant to test rather than presume. We therefore do not apply such a conversion. The band-relative threshold-decision comparison in Section 3.3 is the appropriate cross-study read.

The GENE-UP comparison decomposes into front-end and chemistry components. The principal regulatory comparator in Schmidt et al. [15] is a two-component protocol: a centrifugation-based concentration front end coupled to GENE-UP real-time PCR. The protocol-level performance reported in [15] therefore reflects the joint behavior of both components, not the GENE-UP qPCR chemistry alone. Reading the comparison this way clarifies what the present study can and cannot conclude. What it can conclude is a protocol-level comparison: the centrifugation + GENE-UP protocol evaluated in [15] was reported to require an enrichment step to reach the 1 CFU/g threshold in that dataset, while the Pathotrak + iQ-Check protocol evaluated here operates at that threshold without enrichment, with the gray-zone behavior reported in Section 3. What it cannot conclude is a chemistry-level ranking between GENE-UP qPCR and iQ-Check qPCR, because Schmidt et al. did not include any Bio-Rad qPCR product in their evaluated method set and the two chemistries have not been run on a common dataset. The two qPCR assays operate within the same real-time PCR chemistry family [15]. The substantive implication is that the front end and the chemistry can in principle be varied independently: Pathotrak + GENE-UP is the natural untested cross, and a future head-to-head study could test it directly. The broader detection-agnostic claim across the three Pathotrak studies (digital PCR [12], isothermal amplification [13], real-time PCR here) is summarized in Section 5.

GENE-UP QUANT Salmonella is AOAC-validated for enrichment-free quantification (PTM 061801), with a reported non-enrichment limit of quantification near 10 CFU/mL [15]; the 1 CFU/g level is addressed through adapted protocol options. Our comparison therefore concerns the specific centrifugation-and-GENE-UP protocol and dataset of Schmidt et al., evaluated at the 10 CFU/mL threshold, and is not a statement about the GENE-UP chemistry in general.

### 4.5. Limitations

Several limitations bound the claims above. (i) Strains and chicken sources differed between sites by design. This reflects realistic multi-site implementation conditions. It also precludes attributing any residual site effect specifically to the assay versus to matrix or strain factors. The inoculation strain and its resistance marker and the culture conditions (shaking at USDA and Pathotrak, static at Tyson) also differed by site and are confounded with any site effect. (ii) The Tyson series did not extend above 10 CFU/g, so the per-site Tyson regression is informative about reproducibility within the lower operational band but not about high-concentration linearity. (iii) Cross-method comparison with Schmidt et al. uses one result per rinse from Table 12/Data File A10 (N = 101). This choice avoids pseudo-replication. It also reduces the apparent N relative to repeat-level analyses sometimes reported elsewhere. (iv) Calibration coefficients were estimated on the present multi-site dataset. They have not been independently validated on a held-out external study. The universal IC adjustment should be re-estimated when the dataset is materially extended. (v) Threshold inference uses a latent-truth framework. The framework propagates plating-derived inoculation uncertainty. It assumes a log-normal form for inoculation distributions. It does not propagate uncertainty in the calibration coefficients themselves. (vi) All three sites used the same filter assembly configuration. In a small number of runs the filter was difficult to seat on the bag and part of the rinse volume was lost before loading, which we treat as an execution error rather than a property of the workflow. The magnitude is bounded: because cells partition with the liquid, losing a given fraction of the rinse removes the same fraction of the target, so even a 50% loss corresponds to about one cycle of Cq (≈0.3 log_10_). That displacement is smaller than the half-log gray-zone width, which is consistent with the affected results falling within the expected band. (vii) Overall recovery efficiency across the rinse, concentration, and elution steps was not quantified in this study. The number of target cells reaching an individual qPCR reaction at a given input concentration therefore cannot be stated, and low-copy variance is described qualitatively rather than assigned a value. (viii) This study was designed to evaluate classification at the 1 and 10 CFU/g thresholds rather than to establish a formal limit of detection; detection frequency in Supplementary File S1 was ≥92% at all inoculation levels above 1 CFU/g, and a dedicated LOD/LOQ study remains future work. (ix) Reference concentrations were obtained by 6 × 6 drop-plating at USDA and by spread-plating at Pathotrak and Tyson. Any systematic difference between these methods would enter the latent-truth concentrations and therefore the sensitivity and specificity estimates; agreement between the two was not verified within the study. (x) Reactions from the same inoculation batch (13 batches: USDA four, Pathotrak three, Tyson six) share inoculum, chicken lot, and concentration run and are not independent. The pooled regressions treat them as independent, so their standard errors and *p*-values may be understated; batch identifiers are provided in Supplementary File S1. (xi) Three authors are employed by the manufacturer of the concentrator evaluated here (see Conflicts of Interest); they performed the Pathotrak laboratory arm and took part in study design, analysis, and drafting. The USDA and Tyson arms were run independently by those laboratories, and the raw data and analysis specification (Supplementary File S1; Section 2.8) allow independent re-analysis.

### 4.6. Future Directions

One direction follows naturally from the present results. The USDA intercept shift suggests that recovery-step variants such as backflush volume, air push, eluate handling, etc., are worth a small dedicated study at one site, with the IC-adjusted Cq endpoint as the readout, to convert what is currently a site covariate into a procedure-level recommendation. External validation of the calibration coefficients, including β_IAC_, on an independent dataset remains future work.

A second direction concerns deployment. The same-shift, enrichment-free turnaround discussed in Section 4.1 and Section 4.4 determines where the workflow can be run. Because the workflow requires no incubation step and the concentration procedure is simple and rapid, it is suitable for at-line testing in an on-site plant laboratory equipped for PCR. The current configuration is not intended for continuous in-line monitoring; automation of sample transfer and concentration would be required for that application.

## 5. Conclusions

Together with the two prior Pathotrak papers [12,13], this work establishes the Pathotrak enrichment-free front end as detection-agnostic. The same physical concentration step couples cleanly to digital PCR [12], isothermal amplification [13], and real-time PCR (the present work). In each pairing, detection behavior tracks the intrinsic precision of the downstream platform rather than being limited by the front end.

Within that frame the present study contributes three results. (i) To our knowledge, the first published multi-laboratory quantitative evaluation of the Bio-Rad iQ-Check Salmonella II assay as an enumeration tool in raw poultry. Bio-Rad’s prior published enumeration evidence in this matrix [12] is on the digital PCR platform. The principal published *Salmonella* enumeration comparator in this matrix [15] did not include any Bio-Rad qPCR product. (ii) Completion of the three-paper Pathotrak series across the three real-time PCR-adjacent detection families: digital PCR [12], isothermal amplification [13], and real-time PCR (this study). The real-time PCR family is the chemistry on which the FSIS-adopted GENE-UP platform is built. (iii) Multi-site evaluation across three independent laboratories.

Within this dataset, across sites and strains that also varied by other factors, performance at 10 CFU/g was within the range reported for MPN in a cross-study, band-relative sense (small gray-zone sample sizes; reference studies reporting CFU/mL rather than CFU/g). Turnaround was same-shift and no enrichment step was required. Calibration held across all three sites in slope and IAC coupling after universal IC adjustment. Only a residual intercept shift at the USDA site remained. At 1 CFU/g, the wider gray-zone uncertainty is reported as a measured property of low-copy detection rather than concealed.

The Pathotrak family therefore offers a two-tier methodology. Partition-based pairings (ddPCR [12], nanoplate qPCR) provide absolute quantification in reference-method roles currently occupied by MPN. Real-time qPCR provides routine same-shift threshold screening. The two tiers are complementary. The choice depends on whether the use case calls for absolute quantification or fast threshold-decision screening. Either way, the Pathotrak front end avoids the 6–24 h enrichment incubation required by MPN and ≥5 h enrichment incubation required by published commercial enumeration workflows such as BAX SalQuant. Sample preparation is completed in approximately 30 min and the corresponding result is available within the same shift.

## Figures and Tables

**Figure 1:**
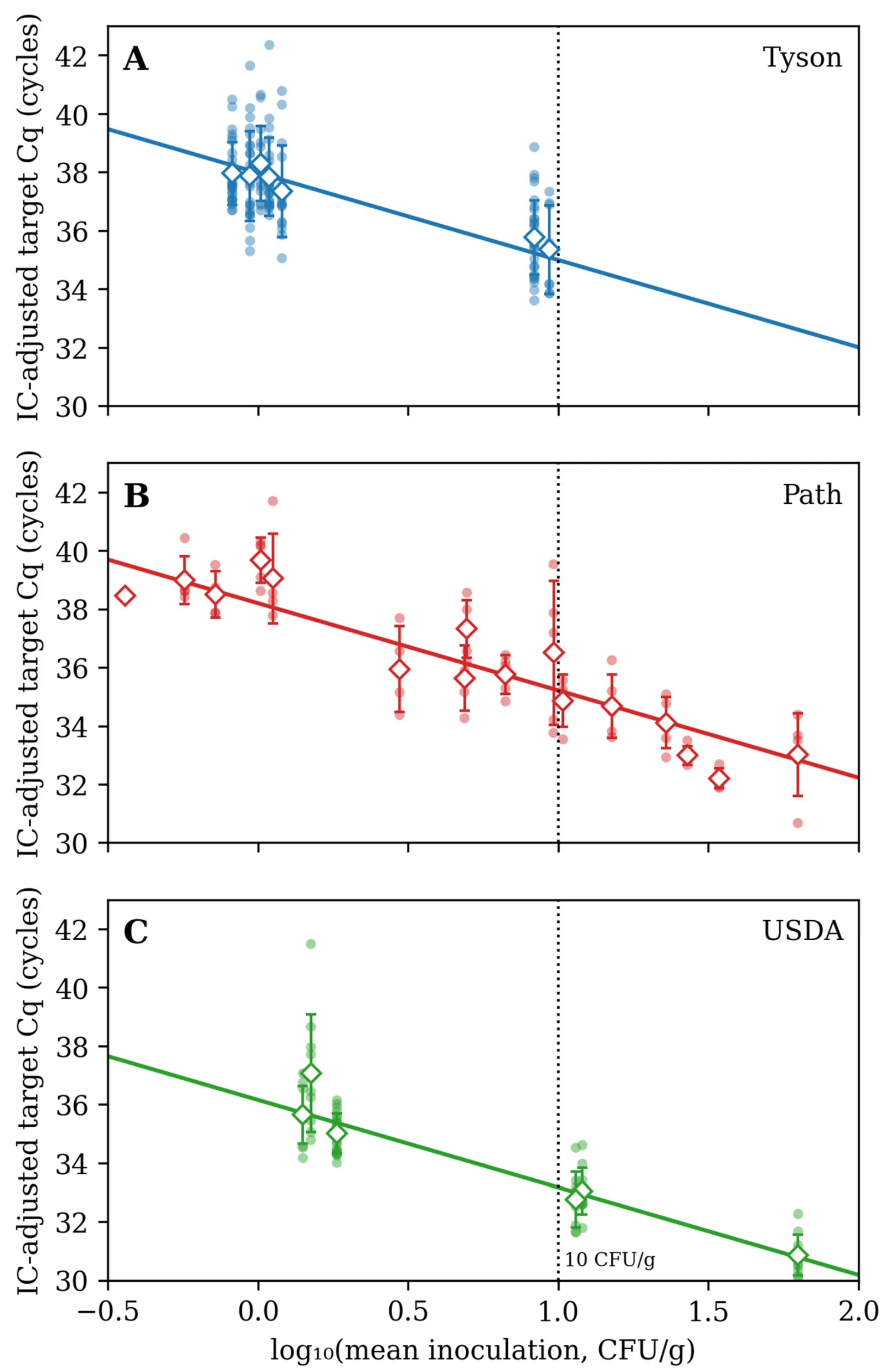
Per-laboratory dose–response after universal internal-control adjustment. Panels show (**A**) Tyson Foods, (**B**) Pathotrak Inc. site, and (**C**) USDA-ARS. Filled circles represent individual detected qPCR reactions; open diamonds with vertical error bars summarize the date × inoculation group means ± 1 SD on the IC-adjusted Cq scale. The solid line in each panel is the lab-effect model fit (pooled OLS with site indicator terms; Pathotrak as reference, see Section 2.8). The dotted vertical line marks the 10 CFU/g decision threshold. All panels share x- and y-axis limits so that the USDA intercept shift is visible by inspection (panel (**C**) sits ≈ 2 cycles below panels (**A**,**B**) at matched inoculation, consistent with the USDA intercept shift of −2.04 cycles reported in Section 3.1).

**Figure 2:**
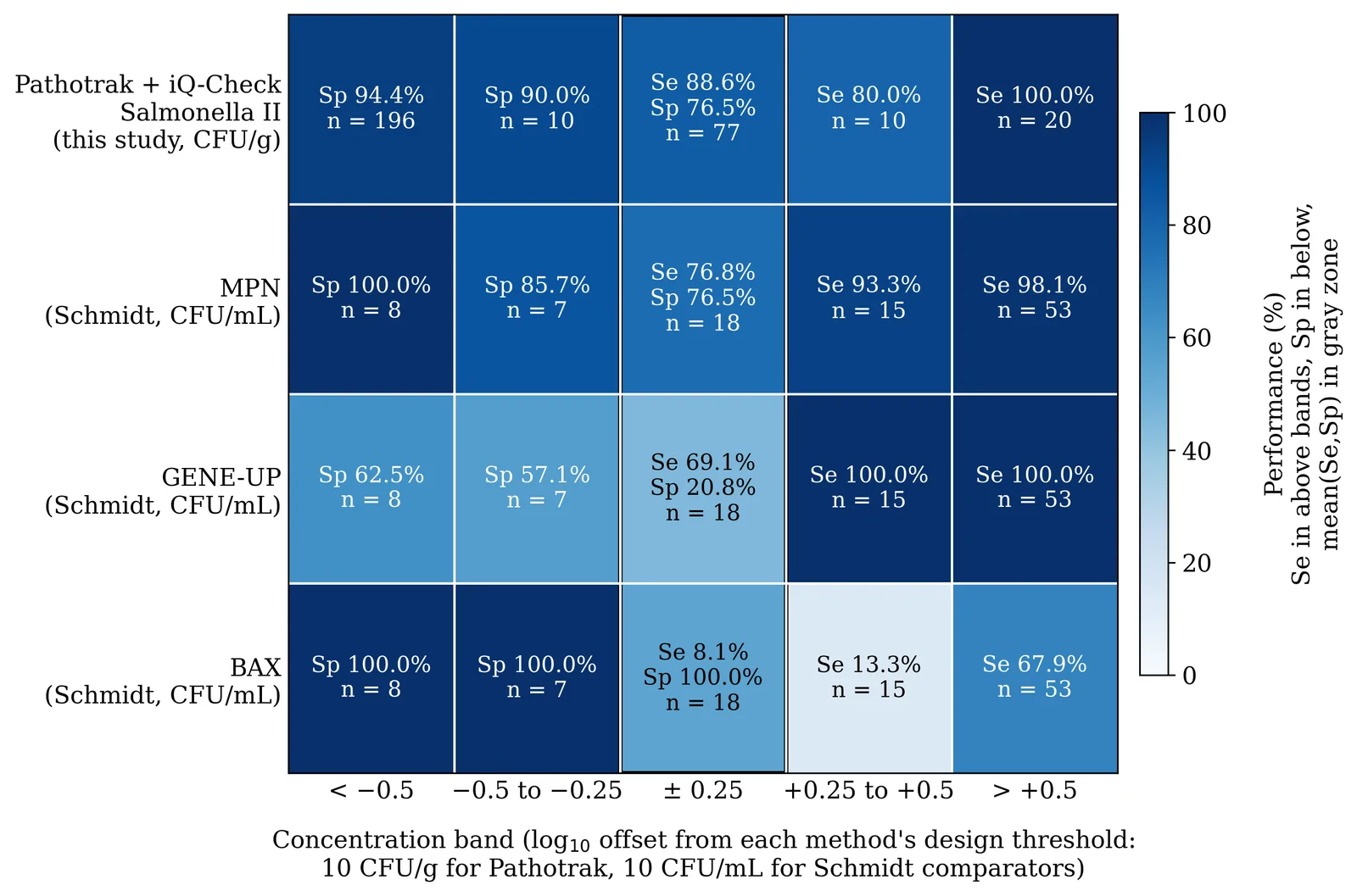
Latent-truth threshold performance at the 10 CFU/g (Pathotrak)/10 CFU/mL (Schmidt et al.) decision threshold for the four enumeration-capable methods. Rows: Pathotrak + iQ-Check Salmonella II (this study, CFU/g of chicken tissue), MPN, GENE-UP, and BAX (Schmidt Table 12/Data File A10, CFU/mL of poultry rinse). Columns are concentration bands defined as fixed log_10_ offsets from each method’s design threshold. Each cell reports maximum-likelihood estimates of sensitivity (Se) and/or specificity (Sp) within the band along with the number of observations (n). Below-threshold bands primarily inform specificity; above-threshold bands primarily inform sensitivity; both are estimated in the gray zone (±0.25 log_10_ around the threshold; 5.62–17.8). Cell shading encodes the displayed metric on a 0–100% scale (Se in above-threshold bands, Sp in below-threshold bands, mean (Se, Sp) in the gray zone), as indicated on the color bar. The gray-zone column is outlined in black. The comparison is band-relative to each method’s design threshold and is not a head-to-head ranking at any fixed absolute concentration.

**Figure 3:**
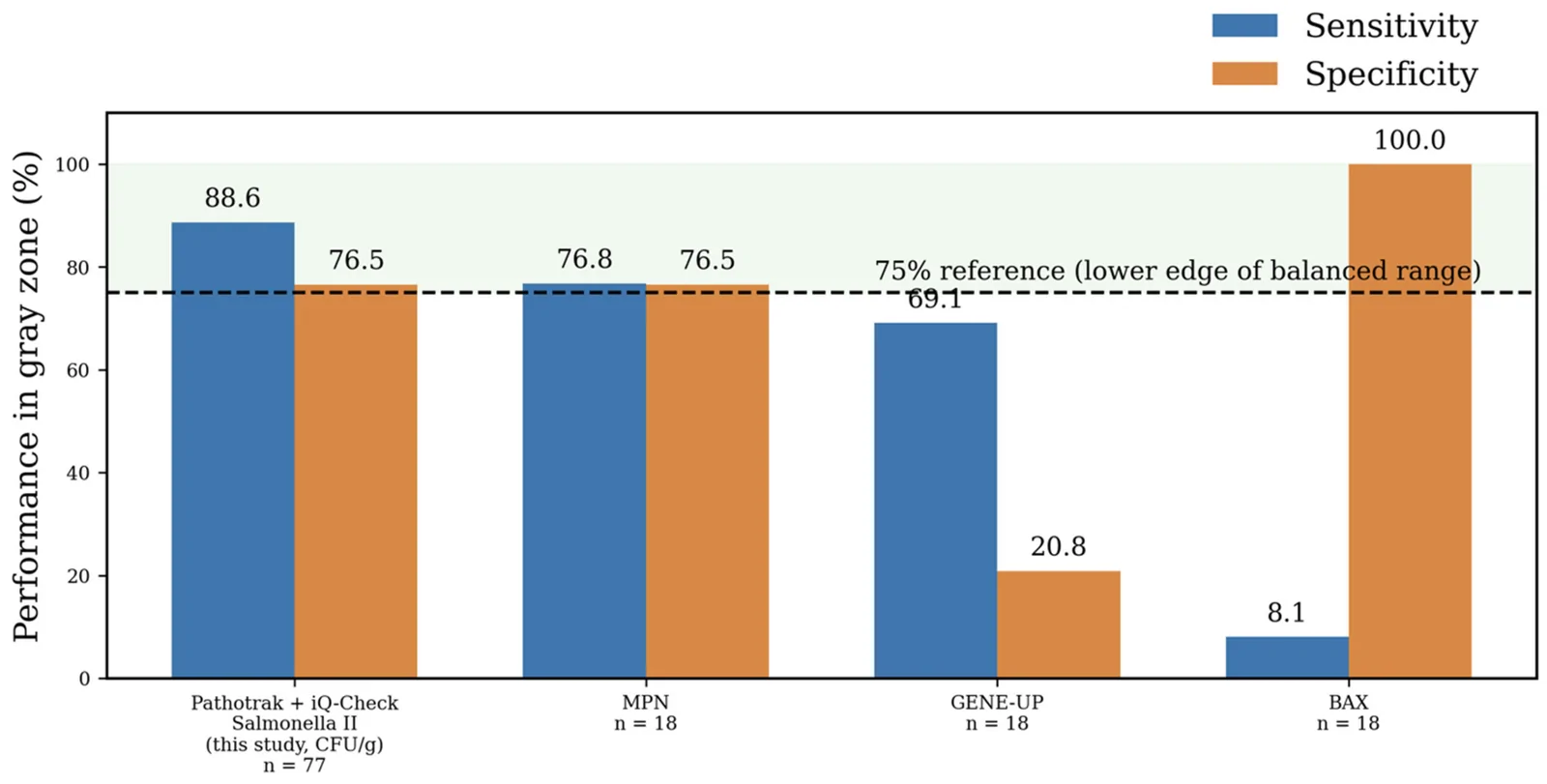
Gray-zone classification balance at the 10 CFU/g decision threshold across four enumeration methods. Sensitivity (Se, blue) and specificity (Sp, orange) within the ±0.25 log_10_ gray zone (5.62–17.8 CFU/g for Pathotrak; 5.62–17.8 CFU/mL for the Schmidt comparator methods) are shown side-by-side. The 75% reference line denotes a pragmatic lower bound for balanced performance (at least 75% on both axes); it is a descriptive comparison aid, not a regulatory or validated acceptance criterion. The dashed line at 75% is a visual reference only. The Pathotrak result is achieved in an enrichment-free, same-shift workflow with sample preparation in approximately 30 min, whereas the MPN comparator and the published commercial enumeration workflows require an enrichment incubation step (6–24 h depending on platform) before the corresponding result is available.

**Figure 4:**
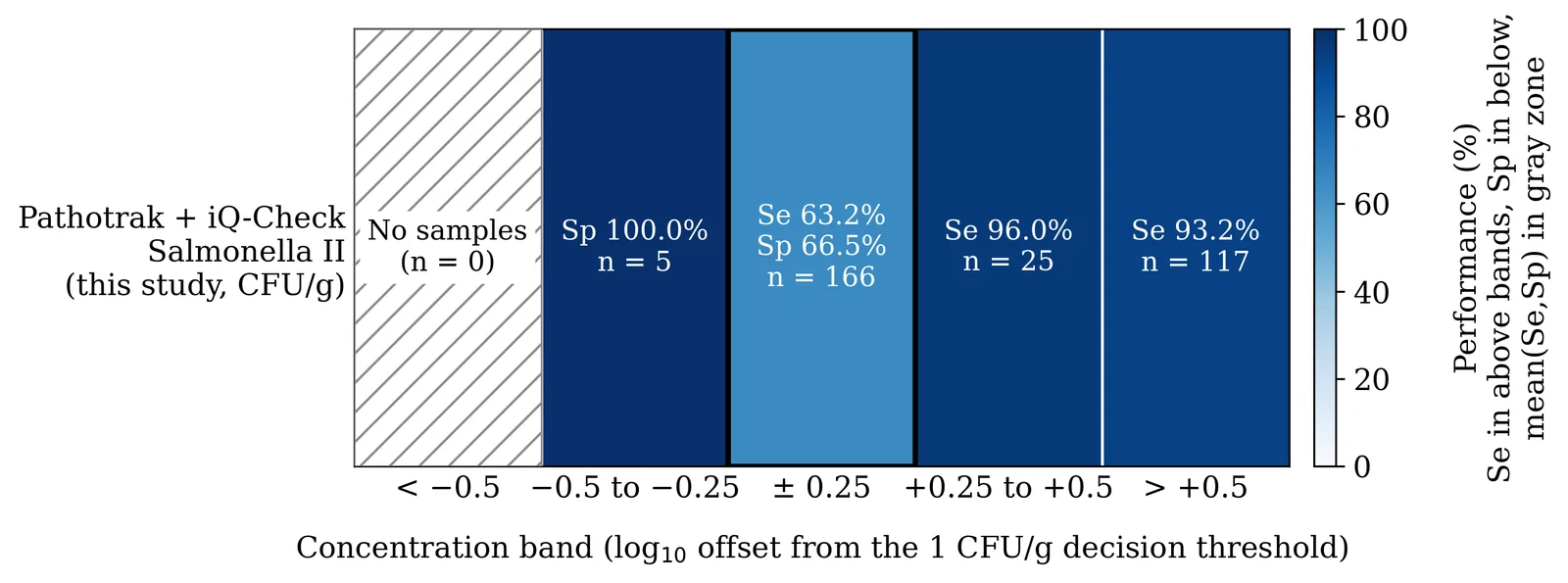
Latent-truth threshold performance at the 1 CFU/g decision threshold (Pathotrak only). Concentration bands are defined as fixed log_10_ offsets from 1 CFU/g; the gray zone (±0.25 log_10_ around 1 CFU/g; 0.562–1.778 CFU/g) is outlined in black. Cell shading and value labels follow the convention used in Figure 2. The far-below band (<0.316 CFU/g) is rendered with diagonal hatching to indicate that no samples were inoculated in this range reflecting study coverage rather than an asymptotic property of the assay (see Section 4.1).

**Table 1:** Descriptive within-laboratory regressions of IC-adjusted target Cq ($Cq_{adj}$) versus mean inoculation. Descriptive ordinary least squares regressions were fit separately within each laboratory using detected reactions only. The predictor is log_10_(mean inoculation) (CFU/g on the original scale). Reported values include number of detected reactions (n), Pearson correlation (r), slope and intercept estimates, $R^{2}$, and RMSE. Non-detect target outcomes (no measurable target Cq by cycle 45) were excluded from regression fits and addressed in threshold analyses.

Laboratory	n (Detected)	Correlation (r)	Intercept	Slope	R^2^	RMSE (Cycles)
Tyson Laboratory Services	142	−0.591	37.850	−2.338	0.350	1.342
Pathotrak Inc. site	74	−0.860	38.559	−3.387	0.739	1.314
USDA	69	−0.846	36.339	−3.125	0.715	1.186

**Table 3:** Between-site detection-call agreement and inoc-adjusted Cq offset within the Tyson × Pathotrak Inc. site overlap window (≤10 CFU/g). Detection rates are tested by Fisher exact. The inoc-adjusted offset is the laboratory term in an OLS regression of IC-adjusted Cq on log_10_(CFU/g) with a laboratory indicator (Pathotrak Inc. as reference; n = 187 detected reactions, R^2^ = 0.376). The 0.50-cycle offset corresponds to approximately 0.2 log_10_ CFU/g, smaller than the half-log gray-zone half-width.

Comparison	Pathotrak Inc. Site	Tyson	Δ	*p*
Detection rate, low bin (≤1.5 CFU/g)	84.0% (n = 25)	82.5% (n = 126)	+1.5 pp	1.00
Detection rate, high bin (5–10 CFU/g)	100.0% (n = 10)	100.0% (n = 38)	0.0 pp	1.00
Inoc-adjusted IC-adjusted Cq offset (Tyson − Path)	—	—	−0.50 cycles (≈0.2 log_10_ CFU/g)	0.033

## Data Availability

The full Pathotrak multi-laboratory dataset analyzed in this study, including 313 qPCR reactions across the Tyson Foods, Pathotrak Inc., and USDA sites, together with the per-figure source data, MLE latent-truth fits, model coefficients, and analysis-ready summary tables, is provided as Supplementary File S1. The Schmidt et al. [15] comparator dataset (Table 12/Data File A10) is available as published Supplementary Material with that work and is reproduced in Supplementary File S1 only as needed to reconstruct the cross-method comparisons reported here.

## Supplementary Materials

The following supporting information can be downloaded at: https://www.mdpi.com/article/10.3390/foods15193441/s1, Supplementary Table S1—cross-method enumeration comparison summary (Schmidt MPN, GENE-UP, BAX; Pathotrak LOLO/LODO; Pathotrak ddPCR 2025); Supplementary Figure S1—latent-truth threshold-10 heatmap for the four PiLOT operating points (PiLOT-95, PiLOT-86, PiLOT-63, PiLOT-50) on the Schmidt Table 12/Data File A10 dataset; and Supplementary File S1—the complete reproducibility package as an Excel workbook, organized into the following tabs: README (orientation, tab index, and figure-to-tab map); Figure_map (mapping of each figure and table to its source tab and fields); Pathotrak_row (per-reaction data for all 313 qPCR reactions across the three sites, including IC-adjusted Cq values and latent-truth fields); Pathotrak_group_means (date × inoculation-level group means underlying Figure 1); Model_coefficients (universal calibration model coefficients); Enumeration_summary_main and Schmidt_A10_plot (within-study and cross-study enumeration metrics underlying Supplementary Table S1); Latent_threshold10_all_methods and Latent_threshold1_Pathotrak (MLE sensitivity and specificity with 95% confidence intervals per concentration band, underlying Figure 2, Figure 3 and Figure 4 and the threshold analyses in Section 3.3, Section 3.4 and Section 4.1); and Threshold10_display_table and Threshold1_display_table (display-format versions of the same fits).
